# Extracellular Vesicles: Opportunities and Challenges for the Treatment of Renal Diseases

**DOI:** 10.3389/fphys.2019.00226

**Published:** 2019-03-19

**Authors:** Tao-Tao Tang, Lin-Li Lv, Hui-Yao Lan, Bi-Cheng Liu

**Affiliations:** ^1^Institute of Nephrology, Zhong Da Hospital, Southeast University School of Medicine, Nanjing, China; ^2^Department of Medicine and Therapeutics, and Li Ka Shing Institute of Health Sciences, The Chinese University of Hong Kong, Hong Kong, Hong Kong

**Keywords:** extracellular vesicles, renal inflammation, renal fibrosis, treatment, drug delivery

## Abstract

Extracellular vesicles (EVs) are lipid-based membrane-bound particles secreted by virtually all types of cells under both physiological and pathological conditions. Given their unique biological and pharmacological properties, EVs have spurred a renewed interest in their utility for therapeutics. Herein, efforts are made to give a comprehensive overview on the recent advances of EV-based therapy in renal diseases. The fact that EVs are implicated in various renal diseases provides us with new therapeutic modalities by eliminating these pathogenic entities. Strategies that target EVs to inhibit their production, release, and uptake will be discussed. Further, EVs-derived predominantly from stem cells can stimulate tissue repair and ameliorate renal injury via transferring proteins and nucleic acids to injured cells. Such EVs can be exploited as agents in renal regenerative medicine. Finally, we will focus on the specific application of EVs as a novel drug delivery system and highlight the challenges of EVs-based therapies for renal diseases.

## Introduction

**Figure 1:**
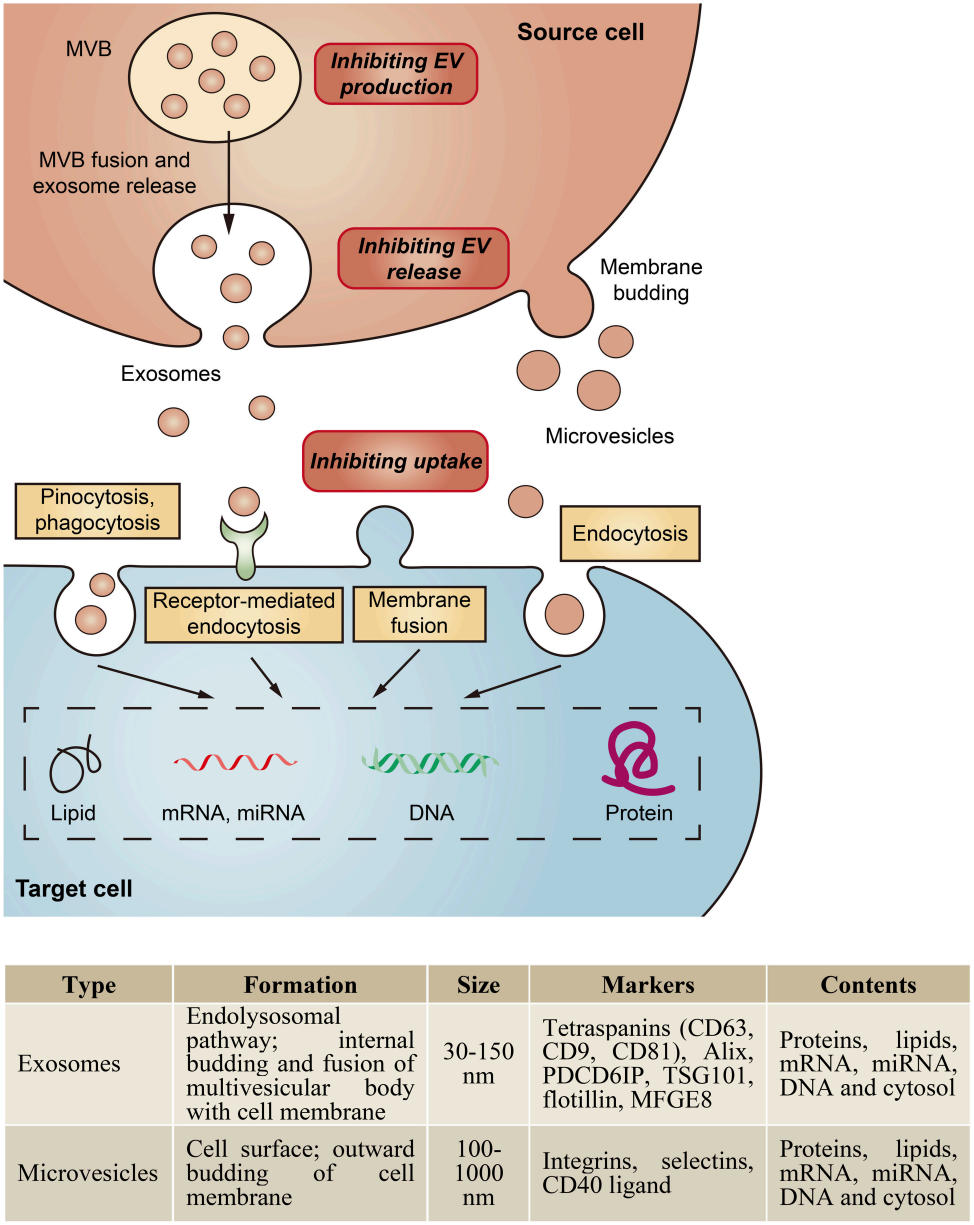
Biogenesis and characteristics of major classes of EVs. EVs can be classed as exosomes, microvesicles, and apoptotic bodies based on their biogenesis and size. Exosomes are formed by the fusion of intracellular multivesicular bodies (MVB) with the plasma membrane, whereas microvesicles are shed directly from the plasma membrane. EVs are taken up by cells by endocytosis, phagocytosis, pinocytosis, or membrane fusion, and subsequently transfer cell membrane receptors or deliver effectors including mRNA, miRNA, DNA, lipid or protein into recipient cells. In addition, EVs could serve as a therapeutic target by inhibition of their production, release or cellular uptake.

Extracellular vesicles (EVs) are small membrane vesicles secreted by various types of cells and are found in most body fluids. Depending on their size and biogenesis, EVs are classified into three major categories: exosomes, microvesicles, and apoptotic bodies (van der Pol et al., 2012; Raposo and Stoorvogel, 2013). Here, we focus on the first two classes of EVs. Exosomes, ranging from 30 to 150 nm in diameter, are formed by the fusion of intracellular multivesicular bodies with the plasma membrane (Colombo et al., 2014), whereas microvesicles, 50–1,000 nm in size, are shed directly from the plasma membrane (Morel et al., 2011; Figure 1).

EVs were initially regarded as cell dust with no biological significance (Wolf, 1967), however, there is growing evidence for their important role not only in the regulation of normal physiological processes, but also in the pathology underlying several diseases (Camussi et al., 2010; Erdbrügger and Le, 2016; Morrison et al., 2016; Zhang W. et al., 2016; Karpman et al., 2017). In kidneys, EVs have been tightly linked to inflammation, fibrosis, thrombosis, adhesion, immune suppression, and growth and regeneration (Zhang W. et al., 2016; Karpman et al., 2017; Li et al., 2019). Therefore, targeting EVs to inhibit their effects should be an emerging strategy for therapy, such as inhibiting EV assembly and release, modifying harmful compositions, and blocking their dissemination and uptake. In addition, as stem cell-derived EVs contain growth factors, proteins, bioactive lipids, and genetic material that can promote tissue repair, they could be utilized directly as therapeutic agents in renal regenerative medicine. For example, EVs from mesenchymal stem cells protected against acute tubular injury and attenuated kidney inflammation (Bruno et al., 2009, 2012; Rani et al., 2015; Eirin et al., 2017).

Finally, given the natural role in transporting bioactive entities of EVs, they also have potential as drug carrier like a “Trojan horse” (van Dommelen et al., 2012; Fuhrmann et al., 2015). Recent studies indicate that EVs can function as efficient carriers of chemotherapeutic drugs (Tang et al., 2012; Yang et al., 2015), RNA drugs (Alvarez-Erviti et al., 2011; Kamerkar et al., 2017) and anti-inflammatory drugs (Sun et al., 2010; Zhuang et al., 2011). In this review, we will focus on recent developments in EV-based therapy as potential targets and as novel therapeutic agents, especially in the use of EVs as smart drug carriers.

## Inhibition of the Release and Uptake of Extracellular Vesicles for Disease Therapy

Within the kidney, EVs can originate from blood cells, endothelial cells, podocytes or tubular epithelial cells (TECs), which have been strongly implicated in the pathogenesis of both acute kidney injury (AKI) and chronic kidney disease (CKD). Our group demonstrated that in the setting of proteinuric kidney disease, albumin triggered TECs to release exosomes packaged with CCL2 mRNA, which was delivered to macrophages and led to interstitial inflammation (Lv et al., 2018a). Borges et al. identified that injured TECs released exosomes containing TGF-β mRNA to activate fibroblasts, contributing to the development of renal fibrosis in post-AKI kidneys (Borges et al., 2013). Moreover, microvesicle-mediated delivery of miR-21 among TECs could also drive the progressive renal fibrosis (Zhou et al., 2013a). Recent data found that transglutaminase-2, a matrix crosslinking enzyme for fibrotic remodeling, was secreted from TECs via exosomes (Furini et al., 2018). Thus, specifically inhibiting the biogenesis or uptake of these pathogenic EVs could be a potential therapeutic approach to alleviate disease progression (Figure 1).

Various cellular components are known to be crucial for the biogenesis and release of EVs, and a number of possible therapeutic targets have been identified. For exosomes, ceramide is an important component in endosomal sorting and exosome biogenesis and its inhibition by GW4869 (neutral sphingomyelinase inhibitor) or amiloride (an antihypertensive agent) decreases exosome production (Trajkovic et al., 2008; Chalmin et al., 2010). GTPases Rab27b can regulate exosome release in some tumor cells, and this was demonstrated to be a therapeutic target (using RNAi) for reducing tumor progression (Ostrowski et al., 2010; Bobrie et al., 2012; Peinado et al., 2012). For microvesicles, the calpain inhibitor calpeptin or calpastain can reduce the shedding of microvesicles (Yano et al., 1993; Zafrani et al., 2012), as well as blocking P2X receptors (Arvidsson et al., 2015). Furthermore, C1 inhibitor lessens the release of endothelial microvesicles, alleviating inflammatory diseases such as vasculitis (Mossberg et al., 2017). However, there are a great many of limitations to target EV biogenesis and release because the precise mechanism remains elusive and is likely to vary among different cells.

In addition to reducing the level of EVs, inhibition of their uptake into cells is also possible by certain substances and antibodies (Mulcahy et al., 2014). Blocking surface phosphatidylserine (which is important for cell adhesion) using diannexin decreases the uptake of EVs derived from tumor cells (Al-Nedawi et al., 2009; Lima et al., 2009). Besides, an antibody to DEL1, annexin V, abciximab, chlorqromazine, cytochalasin D, or cytochalasin B also have been demonstrated to block the uptake of EVs (Barrès et al., 2010; Dasgupta et al., 2012; Faille et al., 2012; Mulcahy et al., 2014), but it is difficult to translate these into therapeutic intervention due to the lack of specific mechanism regarding the key steps in EV trafficking and target definition.

## Intrinsic Therapeutic Potential of Extracellular Vesicles

An increasing number of studies have demonstrated EVs, especially those derived from stem cells, have innate therapeutic potential by virtue of their intrinsic cargoes, such as growth factors, soluble proteins, and nucleic acids (EL Andaloussi et al., 2013). In kidneys, mesenchymal stem cell-derived EVs from different origin exhibit encouraging renoprotective efficacy, as shown in models of AKI, diabetic nephropathy, CKD, and fibrosis. The application of these EVs in kidney diseases has been summarized in Table 1. For instance, Wang et al. showed that exosomes derived from bone marrow MSCs were able to transfer miR-let7c to damaged kidney cells and attenuate renal fibrosis in UUO mice (Wang et al., 2016). Kholia et al. reported that EVs derived from liver stem cells exhibited a regenerative, anti-inflammatory, and anti-fibrotic role in aristolochic acid-induced kidney fibrosis (Kholia et al., 2018). In addition, EVs obtained from umbilical cord MSCs (Zhou et al., 2013b; Ju et al., 2015), Warthon's Jelly MSCs (Zou et al., 2014; Gu et al., 2016; Zhang G. et al., 2016), adipose derived MSCs (Lin et al., 2016; Eirin et al., 2017), kidney MSCs (Choi et al., 2014, 2015; Ranghino et al., 2017), as well as urine derived MSCs (Jiang et al., 2016) also showed potential therapeutic benefits on kidney diseases.

**Table 1:** Therapeutic application of extracellular vesicles in kidney diseases.

**EV origin**	**Kidney injury model**	**EVs doses**	**Injection method**	**Effective molecules**	**References**
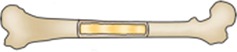	Glycerol-induced AKI	15 μg	Intravenous injection	mRNA	Bruno et al., 2009
		2.2 × 10^8^ EVs	Intravenous injection	miRNA	Collino et al., 2015
BM-MSCs	IRI-induced AKI	200 μg	Renal capsule injection	CCR2 protein	Shen et al., 2016
		30 μg	Intravenous injection	mRNA	Gatti et al., 2011
	Cisplatin-induced AKI	100 μg	Intravenous injection	Not studied	Bruno et al., 2012
	Diabetic nephropathy	5.3 × 10^7^ EVs	Renal subcapsular	Not studied	Nagaishi et al., 2016
	Unilateral ureteral obstruction	1 × 10^6^ EVs	Intravenous injection	miR-let7c	Wang et al., 2016
		30 μg	Intravenous injection	miRNA	Wang et al., 2015
		30 mg	Intravenous injection	miRNA	He et al., 2015
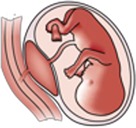	Cisplatin-induced AKI	200 μg	Renal capsule injection	Not studied	Zhou et al., 2013b
	IRI-induced AKI	30 μg	Intravenous injection	HGF mRNA	Ju et al., 2015
UC-MSCs					
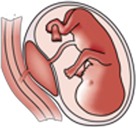	IRI-induced AKI	100 μg	Intravenous injection	Not studied	Zou et al., 2014
		100 μg	Intravenous injection	miR-30	Gu et al., 2016
		100 μg	Intravenous injection	Not studied	Zhang G. et al., 2016
WJ-MSCs					
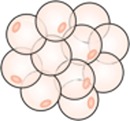	IRI-induced AKI	100 μg	Intravenous injection	Not studied	Lin et al., 2016
	Metabolic syndrome + Renal artery stenosis	1 × 10^10^ EVs	Stenotic renal artery injection	IL-10 protein	Eirin et al., 2017
A-MSCs					
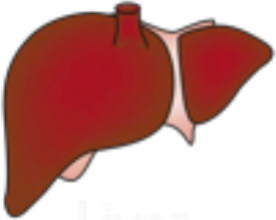	Glycerol-induced AKI	1.88 ± 0.6 × 10^9^ 5.53 ± 2.15 × 10^9^	Intravenous injection	Not studied	Herrera Sanchez et al., 2014
	Aristolochic acid-induced kidney fibrosis	1 × 10^10^ EVs	Intravenous injection	Not studied	Kholia et al., 2018
L-MSCs					
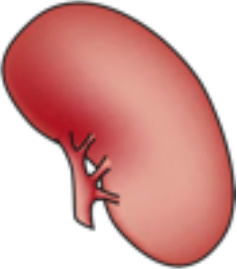	IRI-induced AKI	2 × 10^7^ EVs	Intravenous injection	VEGF, IGF, FGF mRNA	Choi et al., 2014
	IRI-induced AKI	4 × 10^8^ EVs	Intravenous injection	miRNA	Ranghino et al., 2017
	Unilateral ureteral obstruction	2 × 10^7^ EVs	Intravenous injection	mRNA	Choi et al., 2015
K-MSCs					
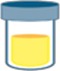	Type I diabetes	100 μg	Intravenous injection	VEGF, TGF-β1, angiogenin and BMP7 protein	Jiang et al., 2016
U-MSCs					
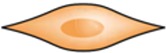	IRI-induced AKI	15 μg	Intravenous injection	Not studied	Burger et al., 2015
	IRI-induced AKI	20 μg	Intravenous injection	miR-486-5p	Viñas et al., 2016
ECFCs					
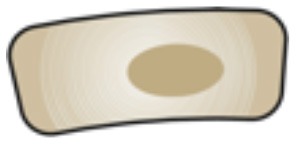	IRI-induced AKI	30 μg	Intravenous injection	miR-126 miR-296	Cantaluppi et al., 2012
	Anti-Thy1.1 glomerulonephritis	30 μg	Intravenous injection	Factor H, CD55, CD59 mRNA	Cantaluppi et al., 2014
EPCs					
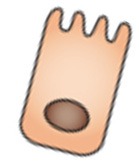	IRI-induced AKI	100 μg	Intravenous injection	mRNA	Dominguez et al., 2017
Hypoxic TECs				
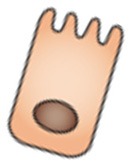	Renal artery stenosis	30 μg	Intravenous injection	mitochondria	Zou et al., 2018
Scattered TECs				

*BM, bone marrow; UC, umbilical cord; WJ, Warthon's Jelly; A, adipose tissue; L, liver; K, kidney; U, urine; ECFC, endothelial colony-forming cells; EPC, endothelial progenitor cell*.

Mechanistically, the protective effect of MSC-EVs on kidney diseases depends on their transfer of genetic materials including mRNA and miRNA (He et al., 2015; Rani et al., 2015; Grange et al., 2017; Nargesi et al., 2017). This was confirmed in many studies when degradation of the RNAs in MSC-EVs using RNase could abolish aforementioned therapeutic benefits (Bruno et al., 2009; Gatti et al., 2011; Choi et al., 2015; Zou et al., 2016), suggesting RNA-dependent biological effects. Also, EVs derived from the Drosha-knockdown MSCs that had miRNA-processing defect showed global downregulation of miRNAs, resulting in ineffective renal repair of glycerol-induced AKI (Collino et al., 2015). Gene ontology analysis further showed that those genes shuttled by MSC-EVs were involved in healing pathways associated with renal regeneration (Collino et al., 2015). Moreover, EVs can also deliver proteins from MSCs to injured kidney cells. Proteins related to cell proliferation, adhesion, migration and morphogenesis have been identified in the vesicles by extensive proteomic analysis (Kim et al., 2012; Jiang et al., 2016; Shen et al., 2016; Eirin et al., 2017). In this regard, an elegant study showed that adipose-derived MSC-EVs attenuated renal inflammation by their cargo of IL-10 in a porcine model of coexisting metabolic syndrome and renal artery stenosis (Eirin et al., 2017).

In addition to MSC-EVs, other sources of cell-derived EVs, such as endothelial colony-forming cells (ECFC), endothelial progenitor cells (EPC), and hypoxic TECs, have shown significant beneficial effects as well (Table 1). In models of ischemic AKI, both ECFC-derived exosomes and EPC-derived EVs ameliorated renal injury via transfer of miRNAs (Cantaluppi et al., 2012; Burger et al., 2015; Viñas et al., 2016). In anti-Thy1.1-induced model of glomerulonephritis, EPC-derived EVs alleviated mesangial cell activation, leukocyte infiltration and apoptosis, which was related to its content of mRNAs coding for anti-apoptotic factors and the complement inhibitors (Cantaluppi et al., 2014). Interestingly, Dominguez et al. found that EVs derived from hypoxic TECs significantly improved renal tubular damage, fibrosis, and microvascular pruning in established renal IRI (Dominguez et al., 2017). However, paradoxically, EVs from injured TECs also contribute to the progression of interstitial inflammation and fibrosis (Borges et al., 2013; Zhou et al., 2013a; Furini et al., 2018; Liu et al., 2018; Lv et al., 2018a; Li et al., 2019), the dual role of TEC-derived EVs need to be further clarified.

## Extracellular Vesicles as Smart Drug Carriers

Currently, the most preferred drug delivery systems are nanoparticle platforms based on liposomes, albumin, polymeric micelles, and nanosized polymer-drug conjugates, which effectively improve the pharmacokinetics and biodistribution of drugs (Kamaly et al., 2016). However, their immunogenicity, stability and toxicity still remain a concern. In this case, EV-based drug delivery—with many of advantages, such as high permeability, less immunogenicity and non-cytotoxicity—appears to be a potential better tolerated and more efficacious alternative, overcoming the limitations observed with nanoparticles (Ha et al., 2016; Lv et al., 2018b; Yang et al., 2018). So far, EVs have been elegantly demonstrated to be therapeutic nanocarriers for delivering a variety of cargos, including siRNAs, miRNAs, proteins, and drugs (van Dommelen et al., 2012; Fuhrmann et al., 2015). But the application of EVs in kidney diseases has just begun.

### Cargo-Loading Techniques

**Figure 2:**
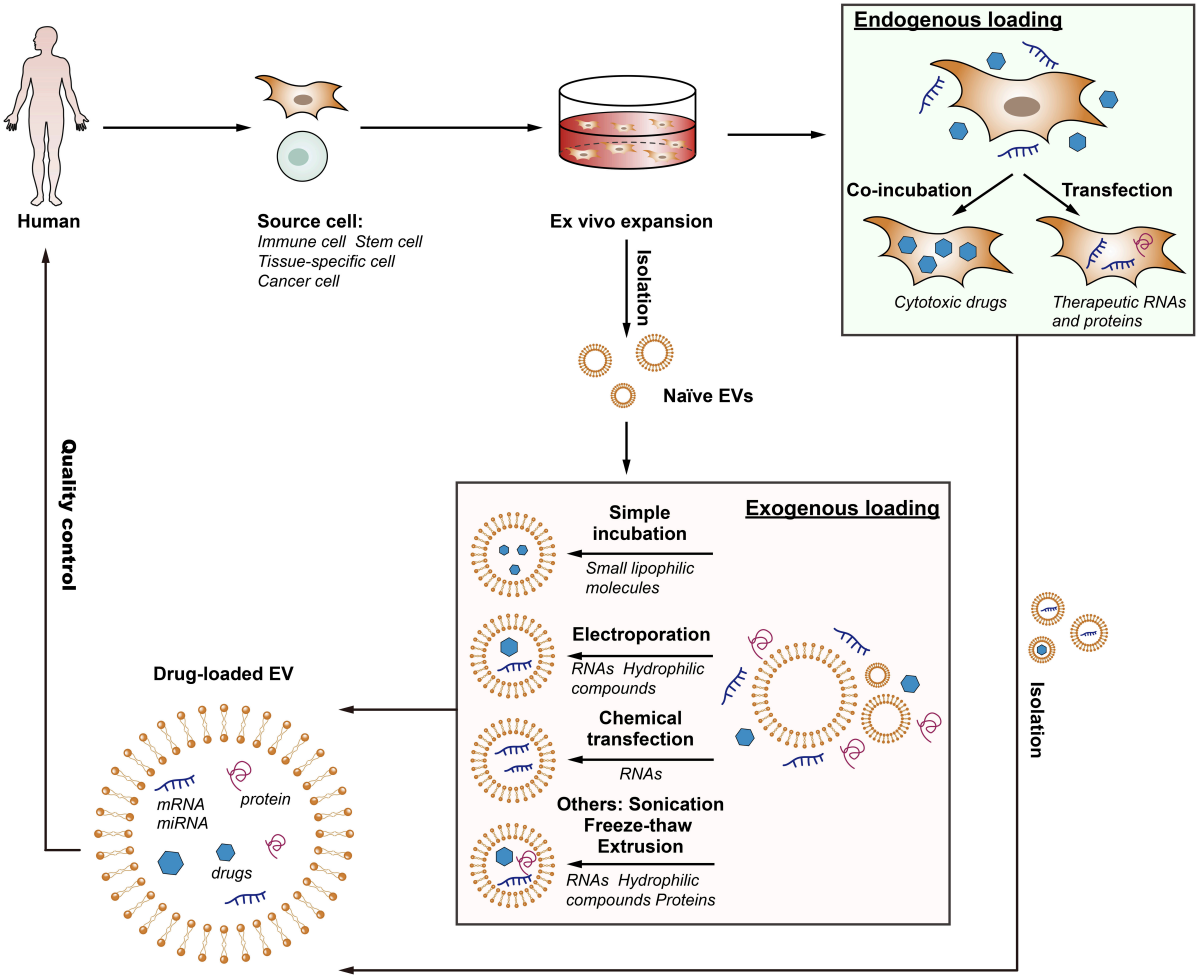
The flow of the production of drug-loaded EVs. EV-based drug delivery requires the correct choice of source cell type for the specific application, and should ideally be patient-derived to avoid triggering immune response. The therapeutic cargo can include different types of siRNA, miRNA, proteins or small molecule compounds such as curcumin or chemotherapeutics. Drug loading can be carried out either endogenously or exogenously. Endogenous loading is achieved by loading source cell with a therapeutic agent or transfecting source cell with drug-encoding gene which is then released in EVs upon collection. Exogenous loading allows the isolation of EVs before their loading with therapeutic cargo with the help of electroporation, simple incubation, chemical transfection, or other approaches. Importantly, the generation process should meet the quality requirements.

In order to employ EV-based drug delivery, it is essential to consider the methods of cargo loading and their suitability under different circumstances. Each loading strategy has its advantages and limitations depending on the type of therapeutic cargo and site of the disease, and thus further understanding is needed to select the optimal approach for mass production. In brief, cargo encapsulation can be performed exogenously or endogenously (van Dommelen et al., 2012; Batrakova and Kim, 2015; Fuhrmann et al., 2015; Figure 2).

#### Endogenously Loading

For endogenously loading, the drug-loaded EVs are isolated from the modified parent cells through genetic engineering or medication with cytotoxic drugs. This way of loading is convenient and requires very few manipulation steps.

##### Transfection

Transfection of donor cells to make them overexpress therapeutic molecules is one of the important ways to load therapeutic RNAs or proteins into EVs (Zeelenberg et al., 2008; Lee et al., 2015; Yim et al., 2016). This method has been used for the loading of miRNAs or proteins in few studies. For example, Ohno et al. transfected HEK293 cells to express GE11 and miR-let7a in order to produce GE11-positive miR-let7a-containing exosomes for targeted treatment of EGFR-expressing tumors (Ohno et al., 2013). Although modification of the parent cells is a feasible and well-developed approach, the productivity of RNA is unstable and the factors influencing the RNA level and RNA loading are not completely clear (Batagov et al., 2011). In addition, peptides fused to N terminus of exosome-associated transmembrane protein may degraded by endosomal proteases, resulting in no detection of the peptide on both cells and exosomes (Hung and Leonard, 2015).

##### Co-incubation

Co-incubation donor cells with exogenous compounds is another approach to load drugs into EVs before they were secreted. It is reported that MSCs-derived exosomes were loaded with paclitaxel by incubating the parent cells with the drug (Pascucci et al., 2014). Similar results were reported for cancer cells that were incubated with different anticancer medicines: doxorubicin, etoposide, carboplatin, irinotecan, epirubicin, and mitoxantrone (Lv et al., 2012; Tang et al., 2012; Yang et al., 2015). However, such technique is of low yield and low entrapment efficacy, and the durg loading is uncontrollable.

#### Exogenously Loading

For exogenously loading, the cargos were packaged into pre-assembled EVs *ex vitro*. A number of methods, including simple incubation, chemical transfection, electroporation and sonication are valid strategies for drug incorporation in this regard (Syn et al., 2017).

##### Simple incubation

Simple incubation is a versatile and feasible approach employed in many cases, through which several small lipophilic molecules, such as curcumin (Sun et al., 2010; Zhuang et al., 2011), doxorubicin (Tian et al., 2014; Rani et al., 2015) and paclitaxel (Yang et al., 2015), are passively loaded into exosomes, but such loading capacity is low and is only work for hydrophobic entities.

##### Chemical transfection

Chemical transfection methods have been reported to load siRNA in EVs by simply incubating exosomes with siRNA-lipofectamine complexes (siRNA embedded in lipid micelles), but the loading efficiency was low compared to electroporation (Shtam et al., 2013). Additionally, the excess of micelles (siRNA embedded in lipid micelles) and the transfection agents (Lipofectamine 2000) were difficult to separate from the exosomes, which may cause immunogenicity and toxicity, limiting its application.

##### Electroporation

Electroporation is an elegant approach that create pores on the EV membrane to allow the penetration of the therapeutic molecules into the EVs, mostly RNAs and hydrophilic compounds (Alvarez-Erviti et al., 2011; Tian et al., 2014; Liao et al., 2018). Alvarez-Erviti et al. loaded siRNA into exosomes successful by electroporation at 400 V and 125 μF, achieving the knockdown of a target protease in Alzheimer's disease (Alvarez-Erviti et al., 2011). Nevertheless, Kooijmans et al. (2013) reported later that electroporation caused extensive siRNA aggregate formation, which could co-precipitate with exosomes by centrifugation and obscured the loading efficacy. Moreover, the pulses may affect the zeta potential and stability of EVs and increase EV aggregation (Syn et al., 2017).

##### Others

Additionally, there are several other methods used for drug loading in EVs, such as sonication, extrusion, and freeze-thaw cycles (Syn et al., 2017; Liao et al., 2018). Sonication is an effective method with high loading efficiency, but is often restricted to the loading of smaller non-biologic molecules. Besides, EV integrity and the loss of intrinsic contents and biological properties after the loading process also deserve further attention.

### Nucleic Acid Delivery

It is known that EVs naturally carry nucleic acids, making them stable in the circulation and protecting from degradation. Given this, EVs may offer unique advantages for genetic therapy, and key studies using EVs as carriers for genetic materials are highlighted below. The first report on EV-mediated transfer of exogenous nucleic acids was published in 2010, when it was shown that THP-1 cells, which were transfected with a miR-150 mimic, secreted miR-150-enriched EVs and that could be functionally delivered to recipient cells (Zhang et al., 2010). A subsequent study conducted by Akao et al. found that THP-1 monocytes transfected with miR-143 mimic *ex vivo* secreted miR-143-containing EVs in nude mice after intravenous injection (Akao et al., 2011). Furthermore, when injected intravenously into UUO mice, engineered MSCs that overexpressed miR-let7c attenuated renal fibrosis via secreting miR-let7c-loaded exosomes (Wang et al., 2016). All these studies have elegantly corroborated the effectiveness of miRNA transfer by EVs.

Small interference RNA (siRNA) is used to inhibit mRNA translation and has great potential for the treatment of a range of diseases. Several studies have been conducted to test the feasibility of using EVs as delivery vehicle for siRNA, and the first study conducted by Alvarez-Erviti et al. found that by expressing a neuron-targeting protein on the surface of exosomes, they could specifically deliver siRNA to the brain resulting in a specific gene knockdown (Alvarez-Erviti et al., 2011). Importantly, the treatment displayed minimal toxicity and immune stimulation, even following repeated administration, suggesting EVs are suitable delivery vectors in RNA interference therapy. This notion has been further confirmed by Wahlgren et al. that the gene MAPK1 was selectively silenced in monocytes and lymphocytes by using siRNA-loaded exosomes derived from human plasma (Wahlgren et al., 2012). More recently, an elegant study employed fibroblast-like mesenchymal cell-derived exosomes to deliver siRNA or short hairpin RNA specific to oncogenic KRAS, achieving enhanced therapeutic efficacy in suppressing tumor growth and improving the overall survival (Kamerkar et al., 2017). Notably, the therapeutic effects of engineered exosomes were greater than siRNA-loaded liposomes (Kamerkar et al., 2017). Beyond miRNA and siRNA delivery, EVs were also exploited to encapsulate adeno-associated viruses (AAVs), which were substantially more efficient than free AAVs for the delivery of genetic cargo into recipient cells (Maguire et al., 2012). Collectively, these studies emphasize the potential of using EVs for the therapeutic delivery of nucleic acids.

### Protein Delivery

In addition to delivering nucleic acids, EVs are also used to deliver large molecules such as proteins. Haney and colleagues found that exosomes loaded with the antioxidant protein catalase (a high molecular weight enzyme, 240 kDa) was successfully delivered across the blood brain barrier (BBB) and provided significant neuroprotective effects in a model of Parkinson's disease (Haney et al., 2015). In this study catalase was incorporated into pre-assembled exosomes *ex vivo* using different methods, and identified sonication and extrusion approaches achieved better loading efficiency, sustained release, and protein preservation (Haney et al., 2015). Similar results were reported by Yuan et al., showing that macrophage-derived exosomes efficiently crossed the BBB and delivered a cargo protein to the brain, further indicating the potency of EVs as nanocarriers for brain delivery of therapeutic proteins (Yuan et al., 2017). The cargo protein in the study was loaded in an exogenous way by mixing with exosomes, in addition, the therapeutic protein can be packaged into EVs by transfecting parental cells as well. For example, HEK-293T cells transfected with suicide gene secreted EVs enriched in suicide mRNA and protein, which were subsequently used to treat Schwannoma tumor in an orthotopic mouse model, leading to reduced tumor growth (Mizrak et al., 2013). Overall, these studies suggest that EVs can serve as novel nanocarriers to effectively deliver therapeutic proteins.

### Drug Delivery

EVs have been utilized as delivery vehicles for therapeutic drugs in extensive research (Sun et al., 2010; Zhuang et al., 2011; Tang et al., 2012; Yang et al., 2015). Early studies demonstrated an anti-inflammatory small molecule compound curcumin could be incorporated into exosomes by mixing curcumin with murine tumor cell line (EL-4) or microglia cell (JSI124)-derived exosomes, and found that exosomal curcumin exhibited enhanced anti-inflammatory activity in LPS-induced septic shock mouse model (Sun et al., 2010; Zhuang et al., 2011). Interestingly, exosomal packaging lead to an increase in the solubility, stability and bioavailability of curcumin (Sun et al., 2010), suggesting EVs are capable to modify the bioavailability of the native drug. For another natural phytochemical compound celastrol, exosome-mediated delivery also improved drug biodistribution and subsequently enhanced its anti-tumor efficacy (Aqil et al., 2016). This study further highlighted the benefits of EVs in enhancing the functionality of drugs, such as solubility, stability and bioavailability.

In addition, the deployment of EVs encapsulating chemotherapeutics such as paclitaxel and doxorubicin has yielded promising results, representing encouraging anti-cancer efficacy with minimal cytotoxicity toward non-cancerous cells (Tang et al., 2012; Jang et al., 2013; Pascucci et al., 2014; Tian et al., 2014; Saari et al., 2015; Toffoli et al., 2015; Yang et al., 2015; Martins-Marques et al., 2016; Srivastava et al., 2016; Syn et al., 2017). For example, anti-cancer drug-loaded exosomes or exosome-like vesicles were shown to traffic to tumor tissue and reduce tumor growth in mice without overt adverse effects (Jang et al., 2013; Tian et al., 2014). Importantly, exosomes had superior therapeutic effects when compared to liposomes (Jang et al., 2013). Moreover, the administration of doxorubicin loaded in exosomes resulted in significantly less drug accumulation in non-target organs and prevented the onset of off-target cardiotoxicity compared with mice treated with unmodified doxorubicin (Saari et al., 2015; Toffoli et al., 2015; Martins-Marques et al., 2016; Srivastava et al., 2016). Thus, the advantages of exosomes packaging may improve the safety profile of cytotoxic agents and present further opportunities to address cancer therapy.

## Benefits and Challenges of Extracellular Vesicle Therapy

Undoubtedly, the field of EV-based therapeutics holds significant promise to enable targeted drug delivery with superior efficiency (Table 2). Compared with existing liposomes or polymeric nanoparticles, the outstanding advantage of EV-based therapy is their naturally lipid and surface protein composition, which enable them to evade phagocytosis, extend blood half-life, and reduce long-term safety issues. For example, CD47 on exosomes can limit their clearance by circulating monocytes (Kamerkar et al., 2017). EVs derived from inflammatory cells expressing integrins or adhesion molecules elicit homing affinities to inflamed tissues (Yuan et al., 2017). The intrinsic contents and biological properties of EVs should be considered and utilized when developing EV-based therapeutics for kidney diseases. Moreover, the small size of EVs facilitates their extravasation, translocation through physical barriers, and passage through extracellular matrix (van den Boorn et al., 2011; van Dommelen et al., 2012). Several studies have demonstrated that EVs successfully cross the BBB and deliver cargos into the brain, but whether EVs are able to pass through the glomerular filtration barrier in healthy states remains unclear. In addition, EVs encapsulation also makes the new drug candidates such as proteins and nucleic acids more stable and targetable to treatment site (Zhu et al., 2012; Bruno et al., 2013).

**Table 2:** Advantages and limitations of extracellular vesicle-based therapy.

**Advantages**	**Limitations**
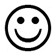 Sanoscale	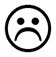 Biochemical composition of EVs unclear
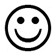 Naturally lipid and surface protein composition	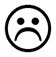 Production or uptake mechanism yet poorly described
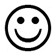 Stable in biological fluids	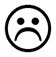 Good manufacturing practice standards lacking
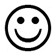 Low immunogenicity	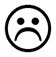 High scale and efficient production difficult
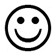 Cell to cell communicators	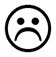 Difficult to package through renal barriers
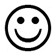 Unidirectional targeting or active targeting by modification	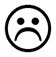 (Pre)clinical evaluation lacking
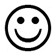 Suitable for multi-drug delivery	
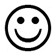 Various drug encapsulation method	
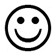 Translocation through physical barriers	

However, before EV-based therapy can be translated to the clinic, several hurdles need to be overcome (Table 2). First, many properties and mechanisms about EV biology such as the biochemical composition of EV currently remain elusive, and the production or uptake mechanism yet poorly described. Even though from the same cell types, EVs may have contradictory effects as a consequence of differences in cell culture conditions, differences in the purification protocols used or due to a lack of robust extracellular vesicle characterization (Zhu et al., 2012; Bruno et al., 2013; EL Andaloussi et al., 2013). In addition, a major bottleneck in the translation of EV-based therapy into clinic is the lack of good manufacturing practice (GMP) standards. To develop clinical-grade EVs, sterile generation, high scale and efficient production of sufficient amounts of EVs with therapeutic payloads for clinical testing are required. Very recently, Mendt and colleagues have illustrated the process and feasibility of generating GMP-grade exosomes (Mendt et al., 2018). Finally, regarding to the particularity of kidney, the glomerular filtration barrier is the primary obstacle that excludes EVs from accessing podocytes or tubular cells (Kamaly et al., 2016). Under normal conditions, only water and small solutes with a molecular weight less than that of albumin (68 kDa) and a hydrodynamic diameter <5–7 nm are allowed to cross the barrier. In disease, the breakdown of the barrier, especially enlarged endothelial gaps, can aid the accumulation of EVs in various kidney cells and components (Kamaly et al., 2016). However, the level of EVs in the kidney is highly restricted based on the degree of injury to the glomerulus. Fortunately, strategies adapted from the nanomedicines, including engineering of the size, shape, and surface charge of EVs, are valid approaches, but how to apply them in EV-based therapy needs to be further investigated.

## Conclusion

**Figure 3:**
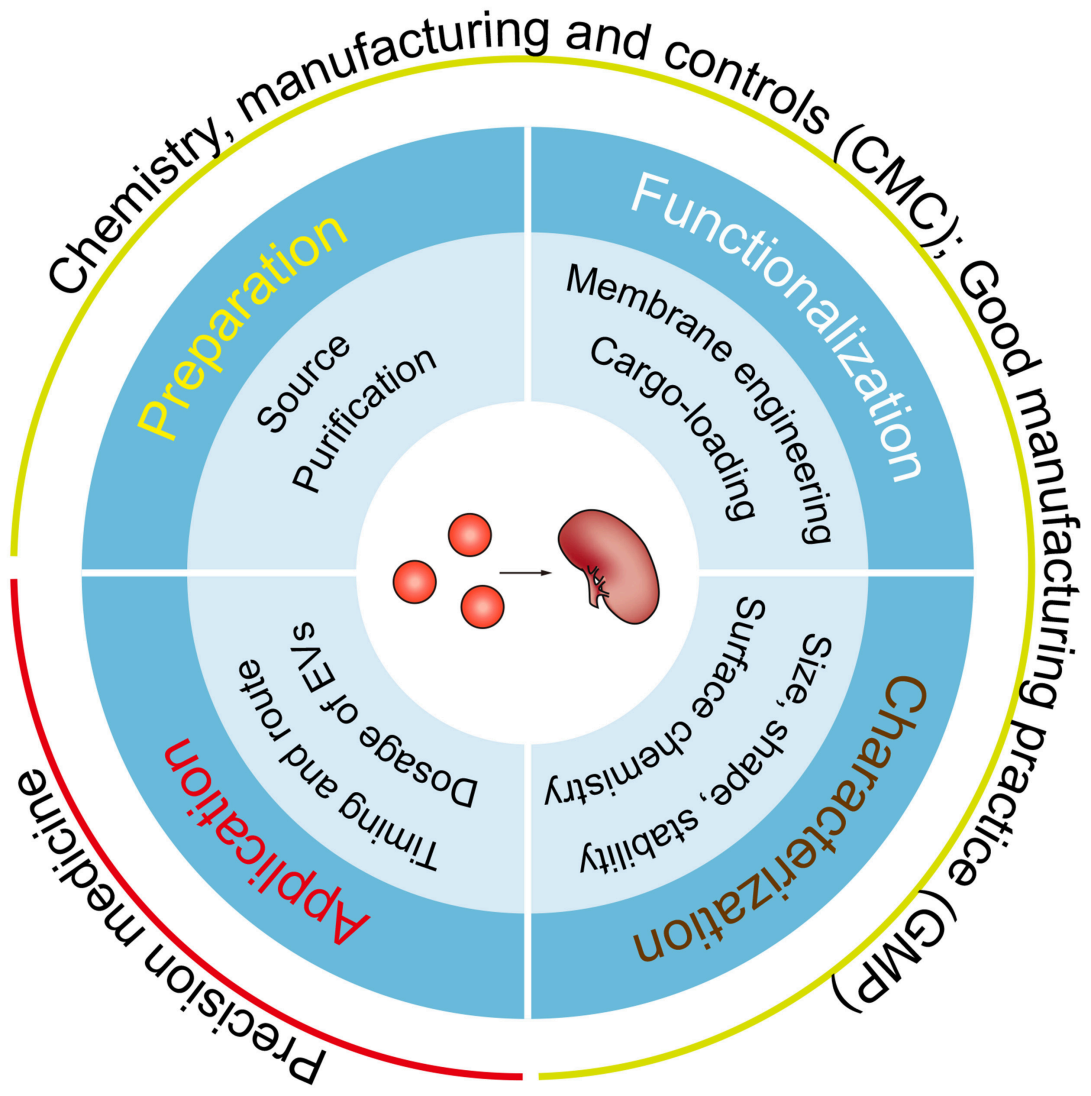
Key considerations for EV-based clinical applications. A number of key steps require optimization before clinical translation, including the source and the purification method of EVs, feasible approaches for membrane engineering and cargo-loading, more comprehensive and precision characterization methods, and the dose, route and timing of EV administration. Of note, all the steps must meet CMC and GMP requirements and the needs of individualized treatment.

The field of EV-based therapy has expanded greatly over the last few years, and application of this strategy to renal disease therapy should have profound translational potential. In this review, we concentrate on emerging therapies including targeting EVs to inhibit their pathogenic effects, exploiting their innate potential for renal regenerative medicine, or using them for robust drug delivery. Although EVs provide an enormous promise and a fresh therapeutic area for nephrotherapy, several key scientific and technical issues need to be urgently addressed, ranging from their preparation, functionalization, and characterization, to their individualized application (Figure 3). Fortunately, numerous studies originally intended for cancer therapy can help us to kick-start the era of EV-based therapy for renal diseases.

## Author Contributions

All authors listed have made a substantial, direct and intellectual contribution to the work, and approved it for publication.

### Conflict of Interest Statement

The authors declare that the research was conducted in the absence of any commercial or financial relationships that could be construed as a potential conflict of interest.

## References

[B1] Akao Y. Iio A. Itoh T. Noguchi S. Itoh Y. Ohtsuki Y.. (2011). Microvesicle-mediated RNA molecule delivery system using monocytes/macrophages. Mol. Ther. 19, 395–399. 10.1038/mt.2010.254 21102562 PMC3034851

[B2] Al-Nedawi K. Meehan B. Kerbel R. S. Allison A. C. Rak J. (2009). Endothelial expression of autocrine VEGF upon the uptake of tumor-derived microvesicles containing oncogenic EGFR. Proc. Natl. Acad. Sci. U.S.A. 106, 3794–3799. 10.1073/pnas.0804543106 19234131 PMC2656159

[B3] Alvarez-Erviti L. Seow Y. Yin H. Betts C. Lakhal S. Wood M. J. (2011). Delivery of siRNA to the mouse brain by systemic injection of targeted exosomes. Nat. Biotechnol. 29, 341–345. 10.1038/nbt.1807 21423189

[B4] Aqil F. Kausar H. Agrawal A. K. Jeyabalan J. Kyakulaga A. H. Munagala R.. (2016). Exosomal formulation enhances therapeutic response of celastrol against lung cancer. Exp. Mol. Pathol. 101, 12–21. 10.1016/j.yexmp.2016.05.013 27235383

[B5] Arvidsson I. Ståhl A. L. Hedström M. M. Kristoffersson A. C. Rylander C. Westman J. S.. (2015). Shiga toxin-induced complement-mediated hemolysis and release of complement-coated red blood cell-derived microvesicles in hemolytic uremic syndrome. J. Immunol. 194, 2309–2318. 10.4049/jimmunol.1402470 25637016

[B6] Barrès C. Blanc L. Bette-Bobillo P. André S. Mamoun R. Gabius H. J.. (2010). Galectin-5 is bound onto the surface of rat reticulocyte exosomes and modulates vesicle uptake by macrophages. Blood 115, 696–705. 10.1182/blood-2009-07-231449 19903899

[B7] Batagov A. O. Kuznetsov V. A. Kurochkin I. V. (2011). Identification of nucleotide patterns enriched in secreted RNAs as putative cis-acting elements targeting them to exosome nano-vesicles. BMC Genomics 12 (Suppl. 3):S18. 10.1186/1471-2164-12-S3-S18 22369587 PMC3333177

[B8] Batrakova E. V. Kim M. S. (2015). Using exosomes, naturally-equipped nanocarriers, for drug delivery. J. Control. Release 219, 396–405. 10.1016/j.jconrel.2015.07.030 26241750 PMC4656109

[B9] Bobrie A. Krumeich S. Reyal F. Recchi C. Moita L. F. Seabra M. C.. (2012). Rab27a supports exosome-dependent and -independent mechanisms that modify the tumor microenvironment and can promote tumor progression. Cancer Res. 72, 4920–4930. 10.1158/0008-5472.CAN-12-0925 22865453

[B10] Borges F. T. Melo S. A. Özdemir B. C. Kato N. Revuelta I. Miller C. A.. (2013). TGF-β1-containing exosomes from injured epithelial cells activate fibroblasts to initiate tissue regenerative responses and fibrosis. J. Am. Soc. Nephrol. 24, 385–392. 10.1681/ASN.2012101031 23274427 PMC3582210

[B11] Bruno S. Collino F. Deregibus M. C. Grange C. Tetta C. Camussi G. (2013). Microvesicles derived from human bone marrow mesenchymal stem cells inhibit tumor growth. Stem Cells Dev. 22, 758–771. 10.1089/scd.2012.0304 23034046

[B12] Bruno S. Grange C. Collino F. Deregibus M. C. Cantaluppi V. Biancone L.. (2012). Microvesicles derived from mesenchymal stem cells enhance survival in a lethal model of acute kidney injury. PLoS ONE 7:e33115. 10.1371/journal.pone.0033115 22431999 PMC3303802

[B13] Bruno S. Grange C. Deregibus M. C. Calogero R. A. Saviozzi S. Collino F.. (2009). Mesenchymal stem cell-derived microvesicles protect against acute tubular injury. J. Am. Soc. Nephrol. 20, 1053–1067. 10.1681/ASN.2008070798 19389847 PMC2676194

[B14] Burger D. Viñas J. L. Akbari S. Dehak H. Knoll W. Gutsol A.. (2015). Human endothelial colony-forming cells protect against acute kidney injury: role of exosomes. Am. J. Pathol. 185, 2309–2323. 10.1016/j.ajpath.2015.04.010 26073035

[B15] Camussi G. Deregibus M. C. Bruno S. Cantaluppi V. Biancone L. (2010). Exosomes/microvesicles as a mechanism of cell-to-cell communication. Kidney Int. 78, 838–848. 10.1038/ki.2010.278 20703216

[B16] Cantaluppi V. Gatti S. Medica D. Figliolini F. Bruno S. Deregibus M. C.. (2012). Microvesicles derived from endothelial progenitor cells protect the kidney from ischemia-reperfusion injury by microRNA-dependent reprogramming of resident renal cells. Kidney Int. 82, 412–427. 10.1038/ki.2012.105 22495296

[B17] Cantaluppi V. Medica D. Mannari C. Stiaccini G. Figliolini F. Dellepiane S.. (2014). Endothelial progenitor cell-derived extracellular vesicles protect from complement-mediated mesangial injury in experimental anti-Thy1.1 glomerulonephritis. Nephrol. Dial. Transplant. 30, 410–422. 10.1093/ndt/gfu364 25488895

[B18] Chalmin F. Ladoire S. Mignot G. Vincent J. Bruchard M. Remy-Martin J. P.. (2010). Membrane-associated Hsp72 from tumor-derived exosomes mediates STAT3-dependent immunosuppressive function of mouse and human myeloid-derived suppressor cells. J. Clin. Invest. 120, 457–471. 10.1172/JCI40483 20093776 PMC2810085

[B19] Choi H. Y. Lee H. G. Kim B. S. Ahn S. H. Jung A. Lee M.. (2015). Mesenchymal stem cell-derived microparticles ameliorate peritubular capillary rarefaction via inhibition of endothelial-mesenchymal transition and decrease tubulointerstitial fibrosis in unilateral ureteral obstruction. Stem Cell Res. Ther. 6:18. 10.1186/s13287-015-0012-6 25889661 PMC4393614

[B20] Choi H. Y. Moon S. J. Ratliff B. B. Ahn S. H. Jung A. Lee M.. (2014). Microparticles from kidney-derived mesenchymal stem cells act as carriers of proangiogenic signals and contribute to recovery from acute kidney injury. PLoS ONE. 9:e87853. 10.1371/journal.pone.0087853 24504266 PMC3913695

[B21] Collino F. Bruno S. Incarnato D. Dettori D. Neri F. Provero P.. (2015). AKI recovery induced by mesenchymal stromal cell-derived extracellular vesicles carrying microRNAs. J. Am. Soc. Nephrol. 26, 2349–2360. 10.1681/ASN.2014070710 25901032 PMC4587694

[B22] Colombo M. Raposo G. Théry C. (2014). Biogenesis, secretion, and intercellular interactions of exosomes and other extracellular vesicles. Annu. Rev. Cell Dev. Biol. 30, 255–289. 10.1146/annurev-cellbio-101512-122326 25288114

[B23] Dasgupta S. K. Le A. Chavakis T. Rumbaut R. E. Thiagarajan P. (2012). Developmental endothelial locus-1 (Del-1) mediates clearance of platelet microparticles by the endothelium. Circulation 125, 1664–1672. 10.1161/CIRCULATIONAHA.111.068833 22388320

[B24] Dominguez J. H. Liu Y. Gao H. Dominguez J. M. II. Xie D.. (2017). Renal tubular cell-derived extracellular vesicles accelerate the recovery of established renal ischemia reperfusion injury. J. Am. Soc. Nephrol. 28, 3533–3544. 10.1681/ASN.2016121278 28747315 PMC5698065

[B25] Eirin A. Zhu X. Y. Puranik A. S. Tang H. McGurren K. A. van Wijnen A. J.. (2017). Mesenchymal stem cell-derived extracellular vesicles attenuate kidney inflammation. Kidney Int. 92, 114–124. 10.1016/j.kint.2016.12.023 28242034 PMC5483390

[B26] EL Andaloussi S. Mäger I. Breakefield X. O. Wood M. J. (2013). Extracellular vesicles: biology and emerging therapeutic opportunities. Nat. Rev. Drug Discov. 12, 347–357. 10.1038/nrd3978 23584393

[B27] Erdbrügger U. Le T. H. (2016). Extracellular vesicles in renal diseases: more than novel biomarkers? J. Am. Soc. Nephrol. 27, 12–26. 10.1681/ASN.2015010074 26251351 PMC4696584

[B28] Faille D. El-Assaad F. Mitchell A. J. Alessi M. C. Chimini G. Fusai T.. (2012). Endocytosis and intracellular processing of platelet microparticles by brain endothelial cells. J. Cell. Mol. Med. 16, 1731–1738. 10.1111/j.1582-4934.2011.01434.x 21883894 PMC3822686

[B29] Fuhrmann G. Herrmann I. K. Stevens M. M. (2015). Cell-derived vesicles for drug therapy and diagnostics: opportunities and challenges. Nano Today 10, 397–409. 10.1016/j.nantod.2015.04.004 28458718 PMC5409525

[B30] Furini G. Schroeder N. Huang L. Boocock D. Scarpellini A. Coveney C.. (2018). Proteomic profiling reveals the transglutaminase-2 externalization pathway in kidneys after unilateral ureteric obstruction. J. Am. Soc. Nephrol. 29, 880–905. 10.1681/ASN.2017050479 29382685 PMC5827594

[B31] Gatti S. Bruno S. Deregibus M. C. Sordi A. Cantaluppi V. Tetta C.. (2011). Microvesicles derived from human adult mesenchymal stem cells protect against ischaemia-reperfusion-induced acute and chronic kidney injury. Nephrol. Dial. Transplant. 26, 1474–1483. 10.1093/ndt/gfr015 21324974

[B32] Grange C. Iampietro C. Bussolati B. (2017). Stem cell extracellular vesicles and kidney injury. Stem Cell Investig. 4:90. 10.21037/sci.2017.11.02 29270416 PMC5723738

[B33] Gu D. Zou X. Ju G. Zhang G. Bao E. Zhu Y. (2016). Mesenchymal stromal cells derived extracellular vesicles ameliorate acute renal ischemia reperfusion injury by inhibition of mitochondrial fission through miR-30. Stem Cells Int. 2016:2093940. 10.1155/2016/2093940 27799943 PMC5069372

[B34] Ha D. Yang N. Nadithe V. (2016). Exosomes as therapeutic drug carriers and delivery vehicles across biological membranes: current perspectives and future challenges. Acta Pharm. Sin. B 6, 287–296. 10.1016/j.apsb.2016.02.001 27471669 PMC4951582

[B35] Haney M. J. Klyachko N. L. Zhao Y. Gupta R. Plotnikova E. G. He Z.. (2015). Exosomes as drug delivery vehicles for Parkinson's disease therapy. J. Control. Release 207, 18–30. 10.1016/j.jconrel.2015.03.033 25836593 PMC4430381

[B36] He J. Wang Y. Lu X. Zhu B. Pei X. Wu J. (2015). Microvesicles derived from bone marrow stem cells protect the kidney both *in vivo* and *in vitro* by microRNA-dependent repairing. Nephrology (Carlton) 20, 591–600. 10.1111/nep.12490 25907000

[B37] Herrera Sanchez M. B. Bruno S. Grange C. Tapparo M. Cantaluppi V. Tetta C.. (2014). Human liver stem cells and derived extracellular vesicles improve recovery in a murine model of acute kidney injury. Stem Cell Res. Ther. 5:124. 10.1186/scrt514 25384729 PMC4446072

[B38] Hung M. E. Leonard J. N. (2015). Stabilization of exosome-targeting peptides via engineered glycosylation. J. Biol. Chem. 290, 8166–8172. 10.1074/jbc.M114.621383 25657008 PMC4375473

[B39] Jang S. C. Kim O. Y. Yoon C. M. Choi D. S. Roh T. Y. Park J.. (2013). Bioinspired exosome-mimetic nanovesicles for targeted delivery of chemotherapeutics to malignant tumors. ACS Nano 7, 7698–7710. 10.1021/nn402232g 24004438

[B40] Jiang Z. Z. Liu Y. M. Niu X. Yin J. Y. Hu B. Guo S. C.. (2016). Exosomes secreted by human urine-derived stem cells could prevent kidney complications from type I diabetes in rats. Stem Cell Res. Ther. 7:24. 10.1186/s13287-016-0287-2 26852014 PMC4744390

[B41] Ju G. Q. Cheng J. Zhong L. Wu S. Zou X. Y. Zhang G. Y.. (2015). Microvesicles derived from human umbilical cord mesenchymal stem cells facilitate tubular epithelial cell dedifferentiation and growth via hepatocyte growth factor induction. PLoS ONE 10:e0121534. 10.1371/journal.pone.0121534 25793303 PMC4368636

[B42] Kamaly N. He J. C. Ausiello D. A. Farokhzad O. C. (2016). Nanomedicines for renal disease: current status and future applications. Nat. Rev. Nephrol. 12, 738–753. 10.1038/nrneph.2016.156 27795549 PMC5593312

[B43] Kamerkar S. LeBleu V. S. Sugimoto H. Yang S. Ruivo C. F. Melo S. A.. (2017). Exosomes facilitate therapeutic targeting of oncogenic KRAS in pancreatic cancer. Nature 546, 498–503. 10.1038/nature22341 28607485 PMC5538883

[B44] Karpman D. Ståhl A. L. Arvidsson I. (2017). Extracellular vesicles in renal disease. Nat. Rev. Nephrol. 13, 545–562. 10.1038/nrneph.2017.98 28736435

[B45] Kholia S. Herrera Sanchez M. B. Cedrino M. Papadimitriou E. Tapparo M. Deregibus M. C.. (2018). Human liver stem cell-derived extracellular vesicles prevent aristolochic acid-induced kidney fibrosis. Front. Immunol. 9:1639. 10.3389/fimmu.2018.01639 30072992 PMC6060249

[B46] Kim H. S. Choi D. Y. Yun S. J. Choi S. M. Kang J. W. Jung J. W.. (2012). Proteomic analysis of microvesicles derived from human mesenchymal stem cells. J. Proteome Res. 11, 839–849. 10.1021/pr200682z 22148876

[B47] Kooijmans S. A. A. Stremersch S. Braeckmans K. de Smedt S. C. Hendrix A. Wood M. J. A.. (2013). Electroporation-induced siRNA precipitation obscures the efficiency of siRNA loading into extracellular vesicles. J. Control Release 172, 229–238. 10.1016/j.jconrel.2013.08.014 23994516

[B48] Lee J. Kim J. Jeong M. Lee H. Goh U. Kim H.. (2015). Liposome-based engineering of cells to package hydrophobic compounds in membrane vesicles for tumor penetration. Nano Lett. 15, 2938–2944. 10.1021/nl5047494 25806671

[B49] Li Z. L. Lv L. L. Tang T. T. Wang B. Feng Y. Zhou L. T.. (2019). HIF-1α inducing exosomal microRNA-23a expression mediates the cross-talk between tubular epithelial cells and macrophages in tubulointerstitial inflammation. Kidney Int. 95, 388–404. 10.1016/j.kint.2018.09.013 30551896

[B50] Liao W. Du Y. Zhang C. Pan F. Yao Y. Zhang T.. (2018). Exosomes: the next generation of endogenous nanomaterials for advanced drug delivery and therapy. Acta Biomater. 86, 1–14. 10.1016/j.actbio.2018.12.045 30597259

[B51] Lima L. G. Chammas R. Monteiro R. Q. Moreira M. E. Barcinski M. A. (2009). Tumor-derived microvesicles modulate the establishment of metastatic melanoma in a phosphatidylserine-dependent manner. Cancer Lett. 283, 168–175. 10.1016/j.canlet.2009.03.041 19401262

[B52] Lin K. C. Yip H. K. Shao P. L. Wu S. C. Chen K. H. Chen Y. T.. (2016). Combination of adipose-derived mesenchymal stem cells (ADMSC) and ADMSC-derived exosomes for protecting kidney from acute ischemia-reperfusion injury. Int. J. Cardiol. 216, 173–185. 10.1016/j.ijcard.2016.04.061 27156061

[B53] Liu B. C. Tang T. T. Lv L. L. Lan H. Y. (2018). Renal tubule injury: a driving force toward chronic kidney disease. Kidney Int. 93, 568–579. 10.1016/j.kint.2017.09.033 29361307

[B54] Lv L. H. Wan Y. L. Lin Y. Zhang W. Yang M. Li G. L.. (2012). Anticancer drugs cause release of exosomes with heat shock proteins from human hepatocellular carcinoma cells that elicit effective natural killer cell antitumor responses *in vitro*. J. Biol. Chem. 287, 15874–15885. 10.1074/jbc.M112.340588 22396543 PMC3346092

[B55] Lv L. L. Feng Y. Wen Y. Wu W. J. Ni H. F. Li Z. L.. (2018a). Exosomal CCL2 from tubular epithelial cells is critical for albumin-induced tubulointerstitial inflammation. J. Am. Soc. Nephrol. 29, 919–935. 10.1681/ASN.2017050523 29295871 PMC5827595

[B56] Lv L. L. Wu W. J. Feng Y. Li Z. L. Tang T. T. Liu B. C. (2018b). Therapeutic application of extracellular vesicles in kidney disease: promises and challenges. J. Cell. Mol. Med. 22, 728–737. 10.1111/jcmm.13407 29083099 PMC5783839

[B57] Maguire C. A. Balaj L. Sivaraman S. Crommentuijn M. H. Ericsson M. Mincheva-Nilsson L.. (2012). Microvesicle-associated AAV vector as a novel gene delivery system. Mol. Ther. 20, 960–971. 10.1038/mt.2011.303 22314290 PMC3345986

[B58] Martins-Marques T. Pinho M. J. Zuzarte M. Oliveira C. Pereira P. Sluijter J. P.. (2016). Presence of Cx43 in extracellular vesicles reduces the cardiotoxicity of the anti-tumour therapeutic approach with doxorubicin. J. Extracell. Vesicles 5:32538. 10.3402/jev.v5.32538 27702427 PMC5045474

[B59] Mendt M. Kamerkar S. Sugimoto H. McAndrews K. M. Wu C. C. Gagea M.. (2018). Generation and testing of clinical-grade exosomes for pancreatic cancer. JCI Insight 3:99263. 10.1172/jci.insight.99263 29669940 PMC5931131

[B60] Mizrak A. Bolukbasi M. F. Ozdener G. B. Brenner G. J. Madlener S. Erkan E. P.. (2013). Genetically engineered microvesicles carrying suicide mRNA/protein inhibit schwannoma tumor growth. Mol. Ther. 21, 101–108. 10.1038/mt.2012.161 22910294 PMC3538300

[B61] Morel O. Jesel L. Freyssinet J. M. Toti F. (2011). Cellular mechanisms underlying the formation of circulating microparticles. Arterioscler. Thromb. Vasc. Biol. 31, 15–26. 10.1161/ATVBAHA.109.200956 21160064

[B62] Morrison E. E. Bailey M. A. Dear J. W. (2016). Renal extracellular vesicles: from physiology to clinical application. J. Physiol. 594, 5735–5748. 10.1113/JP272182 27104781 PMC5063943

[B63] Mossberg M. Ståhl A. L. Kahn R. Kristoffersson A. C. Tati R. Heijl C.. (2017). C1-Inhibitor decreases the release of vasculitis-like chemotactic endothelial microvesicles. J. Am. Soc. Nephrol. 28, 2472–2481. 10.1681/ASN.2016060637 28289183 PMC5533224

[B64] Mulcahy L. A. Pink R. C. Carter D. R. (2014). Routes and mechanisms of extracellular vesicle uptake. J. Extracell. Vesicles 3:24641. 10.3402/jev.v3.24641 25143819 PMC4122821

[B65] Nagaishi K. Mizue Y. Chikenji T. Otani M. Nakano M. Konari N.. (2016). Mesenchymal stem cell therapy ameliorates diabetic nephropathy via the paracrine effect of renal trophic factors including exosomes. Sci. Rep. 6:34842. 10.1038/srep34842 27721418 PMC5056395

[B66] Nargesi A. A. Lerman L. O. Eirin A. (2017). Mesenchymal stem cell-derived extracellular vesicles for renal repair. Curr. Gene Ther. 17, 29–42. 10.2174/1566523217666170412110724 28403795 PMC5628022

[B67] Ohno S. Takanashi M. Sudo K. Ueda S. Ishikawa A. Matsuyama N.. (2013). Systemically injected exosomes targeted to EGFR deliver antitumor microRNA to breast cancer cells. Mol. Ther. 21, 185–191. 10.1038/mt.2012.180 23032975 PMC3538304

[B68] Ostrowski M. Carmo N. B. Krumeich S. Fanget I. Raposo G. Savina A.. (2010). Rab27a and Rab27b control different steps of the exosome secretion pathway. Nat. Cell Biol. 12, 19–30. 10.1038/ncb2000 19966785

[B69] Pascucci L. Coccè V. Bonomi A. Ami D. Ceccarelli P. Ciusani E.. (2014). Paclitaxel is incorporated by mesenchymal stromal cells and released in exosomes that inhibit *in vitro* tumor growth: a new approach for drug delivery. J. Control. Release 192, 262–270. 10.1016/j.jconrel.2014.07.042 25084218

[B70] Peinado H. Alečković M. Lavotshkin S. Matei I. Costa-Silva B. Moreno-Bueno G.. (2012). Melanoma exosomes educate bone marrow progenitor cells toward a pro-metastatic phenotype through MET. Nat. Med. 18, 883–891. 10.1038/nm.2753 22635005 PMC3645291

[B71] Ranghino A. Bruno S. Bussolati B. Moggio A. Dimuccio V. Tapparo M.. (2017). The effects of glomerular and tubular renal progenitors and derived extracellular vesicles on recovery from acute kidney injury. Stem Cell Res. Ther. 8:24. 10.1186/s13287-017-0478-5 28173878 PMC5297206

[B72] Rani S. Ryan A. E. Griffin M. D. Ritter T. (2015). Mesenchymal stem cell-derived extracellular vesicles: toward cell-free therapeutic applications. Mol. Ther. 23, 812–823. 10.1038/mt.2015.44 25868399 PMC4427881

[B73] Raposo G. Stoorvogel W. (2013). Extracellular vesicles: exosomes, microvesicles, and friends. J. Cell Biol. 200, 373–383. 10.1083/jcb.201211138 23420871 PMC3575529

[B74] Saari H. Lázaro-Ibáñez E. Viitala T. Vuorimaa-Laukkanen E. Siljander P. Yliperttula M. (2015). Microvesicle- and exosome-mediated drug delivery enhances the cytotoxicity of Paclitaxel in autologous prostate cancer cells. J. Control. Release 220, 727–737. 10.1016/j.jconrel.2015.09.031 26390807

[B75] Shen B. Liu J. Zhang F. Wang Y. Qin Y. Zhou Z.. (2016). CCR2 positive exosome released by mesenchymal stem cells suppresses macrophage functions and alleviates ischemia/reperfusion-induced renal injury. Stem Cells Int. 2016:1240301. 10.1155/2016/1240301 27843457 PMC5098097

[B76] Shtam T. A. Kovalev R. A. Varfolomeeva E. Y. Makarov E. M. Kil Y. V. Filatov M. V. (2013). Exosomes are natural carriers of exogenous siRNA to human cells *in vitro*. Cell Commun. Signal. 11:88. 10.1186/1478-811X-11-88 24245560 PMC3895799

[B77] Srivastava A. Amreddy N. Babu A. Panneerselvam J. Mehta M. Muralidharan R.. (2016). Nanosomes carrying doxorubicin exhibit potent anticancer activity against human lung cancer cells. Sci. Rep. 6:38541. 10.1038/srep38541 27941871 PMC5150529

[B78] Sun D. Zhuang X. Xiang X. Liu Y. Zhang S. Liu C.. (2010). A novel nanoparticle drug delivery system: the anti-inflammatory activity of curcumin is enhanced when encapsulated in exosomes. Mol. Ther. 18, 1606–1614. 10.1038/mt.2010.105 20571541 PMC2956928

[B79] Syn N. L. Wang L. Chow E. K. Lim C. T. Goh B. C. (2017). Exosomes in cancer nanomedicine and immunotherapy: prospects and challenges. Trends Biotechnol. 35, 665–676. 10.1016/j.tibtech.2017.03.004 28365132

[B80] Tang K. Zhang Y. Zhang H. Xu P. Liu J. Ma J.. (2012). Delivery of chemotherapeutic drugs in tumour cell-derived microparticles. Nat. Commun. 3:1282. 10.1038/ncomms2282 23250412

[B81] Tian Y. Li S. Song J. Ji T. Zhu M. Anderson G. J.. (2014). A doxorubicin delivery platform using engineered natural membrane vesicle exosomes for targeted tumor therapy. Biomaterials 35, 2383–2390. 10.1016/j.biomaterials.2013.11.083 24345736

[B82] Toffoli G. Hadla M. Corona G. Caligiuri I. Palazzolo S. Semeraro S.. (2015). Exosomal doxorubicin reduces the cardiac toxicity of doxorubicin. Nanomedicine 10, 2963–2971. 10.2217/nnm.15.118 26420143

[B83] Trajkovic K. Hsu C. Chiantia S. Rajendran L. Wenzel D. Wieland F.. (2008). Ceramide triggers budding of exosome vesicles into multivesicular endosomes. Science 319, 1244–1247. 10.1126/science.1153124 18309083

[B84] van den Boorn J. G. Schlee M. Coch C. Hartmann G. (2011). SiRNA delivery with exosome nanoparticles. Nat. Biotechnol. 29, 325–326. 10.1038/nbt.1830 21478846

[B85] van der Pol E. Böing A. N. Harrison P. Sturk A. Nieuwland R. (2012). Classification, functions, and clinical relevance of extracellular vesicles. Pharmacol. Rev. 64, 676–705. 10.1124/pr.112.005983 22722893

[B86] van Dommelen S. M. Vader P. Lakhal S. Kooijmans S. A. van Solinge W. W. Wood M. J.. (2012). Microvesicles and exosomes: opportunities for cell-derived membrane vesicles in drug delivery. J. Control. Release 161, 635–644. 10.1016/j.jconrel.2011.11.021 22138068

[B87] Viñas J. L. Burger D. Zimpelmann J. Haneef R. Knoll W. Campbell P.. (2016). Transfer of microRNA-486-5p from human endothelial colony forming cell-derived exosomes reduces ischemic kidney injury. Kidney Int. 90, 1238–1250. 10.1016/j.kint.2016.07.015 27650731

[B88] Wahlgren J. De L Karlson T. Brisslert M. Vaziri Sani F. Telemo E. Sunnerhagen P.. (2012). Plasma exosomes can deliver exogenous short interfering RNA to monocytes and lymphocytes. Nucleic Acids Res. 40:e130. 10.1093/nar/gks463 22618874 PMC3458529

[B89] Wang B. Yao K. Huuskes B. M. Shen H. H. Zhuang J. Godson C.. (2016). Mesenchymal stem cells deliver exogenous microRNA-let7c via exosomes to attenuate renal fibrosis. Mol. Ther. 24, 1290–1301. 10.1038/mt.2016.90 27203438 PMC5088767

[B90] Wang Y. Lu X. He J. Zhao W. (2015). Influence of erythropoietin on microvesicles derived from mesenchymal stem cells protecting renal function of chronic kidney disease. Stem Cell Res. Ther. 6:100. 10.1186/s13287-015-0095-0 25998259 PMC4469245

[B91] Wolf P. (1967). The nature and significance of platelet products in human plasma. Br. J. Haematol. 13, 269–288. 10.1111/j.1365-2141.1967.tb08741.x 6025241

[B92] Yang B. Chen Y. Shi J. (2018). Exosome biochemistry and advanced nanotechnology for next-generation theranostic platforms. Adv. Mater. Weinheim. 20:e1802896 10.1002/adma.201802896 30126052

[B93] Yang T. Martin P. Fogarty B. Brown A. Schurman K. Phipps R.. (2015). Exosome delivered anticancer drugs across the blood-brain barrier for brain cancer therapy in Danio rerio. Pharm. Res. 32, 2003–2014. 10.1007/s11095-014-1593-y 25609010 PMC4520542

[B94] Yano Y. Shiba E. Kambayashi J. Sakon M. Kawasaki T. Fujitani K.. (1993). The effects of calpeptin (a calpain specific inhibitor) on agonist induced microparticle formation from the platelet plasma membrane. Thromb. Res. 71, 385–396. 10.1016/0049-3848(93)90163-I 8236165

[B95] Yim N. Ryu S. W. Choi K. Lee K. R. Lee S. Choi H.. (2016). Exosome engineering for efficient intracellular delivery of soluble proteins using optically reversible protein-protein interaction module. Nat. Commun. 7:12277. 10.1038/ncomms12277 27447450 PMC4961865

[B96] Yuan D. Zhao Y. Banks W. A. Bullock K. M. Haney M. Batrakova E.. (2017). Macrophage exosomes as natural nanocarriers for protein delivery to inflamed brain. Biomaterials 142, 1–12. 10.1016/j.biomaterials.2017.07.011 28715655 PMC5603188

[B97] Zafrani L. Gerotziafas G. Byrnes C. Hu X. Perez J. Lévi C.. (2012). Calpastatin controls polymicrobial sepsis by limiting procoagulant microparticle release. Am. J. Respir. Crit. Care Med. 185, 744–755. 10.1164/rccm.201109-1686OC 22268136 PMC3326423

[B98] Zeelenberg I. S. Ostrowski M. Krumeich S. Bobrie A. Jancic C. Boissonnas A.. (2008). Targeting tumor antigens to secreted membrane vesicles *in vivo* induces efficient antitumor immune responses. Cancer Res. 68, 1228–1235. 10.1158/0008-5472.CAN-07-3163 18281500

[B99] Zhang G. Zou X. Huang Y. Wang F. Miao S. Liu G.. (2016). Mesenchymal stromal cell-derived extracellular vesicles protect against acute kidney injury through anti-oxidation by enhancing Nrf2/ARE activation in rats. Kidney Blood Press. Res. 41, 119–128. 10.1159/000443413 26894749

[B100] Zhang W. Zhou X. Zhang H. Yao Q. Liu Y. Dong Z. (2016). Extracellular vesicles in diagnosis and therapy of kidney diseases. Am. J. Physiol. Renal Physiol. 311, F844–F851. 10.1152/ajprenal.00429.2016 27582107 PMC5130456

[B101] Zhang Y. Liu D. Chen X. Li J. Li L. Bian Z.. (2010). Secreted monocytic miR-150 enhances targeted endothelial cell migration. Mol. Cell 39, 133–144. 10.1016/j.molcel.2010.06.010 20603081

[B102] Zhou Y. Xiong M. Fang L. Jiang L. Wen P. Dai C.. (2013a). miR-21-containing microvesicles from injured tubular epithelial cells promote tubular phenotype transition by targeting PTEN protein. Am. J. Pathol. 183, 1183–1196. 10.1016/j.ajpath.2013.06.032 23978520

[B103] Zhou Y. Xu H. Xu W. Wang B. Wu H. Tao Y.. (2013b). Exosomes released by human umbilical cord mesenchymal stem cells protect against cisplatin-induced renal oxidative stress and apoptosis *in vivo* and *in vitro*. Stem Cell Res. Ther. 4:34. 10.1186/scrt194 23618405 PMC3707035

[B104] Zhu W. Huang L. Li Y. Zhang X. Gu J. Yan Y.. (2012). Exosomes derived from human bone marrow mesenchymal stem cells promote tumor growth *in vivo*. Cancer Lett. 315, 28–37. 10.1016/j.canlet.2011.10.002 22055459

[B105] Zhuang X. Xiang X. Grizzle W. Sun D. Zhang S. Axtell R. C.. (2011). Treatment of brain inflammatory diseases by delivering exosome encapsulated anti-inflammatory drugs from the nasal region to the brain. Mol. Ther. 19, 1769–1779. 10.1038/mt.2011.164 21915101 PMC3188748

[B106] Zou X. Gu D. Xing X. Cheng Z. Gong D. Zhang G.. (2016). Human mesenchymal stromal cell-derived extracellular vesicles alleviate renal ischemic reperfusion injury and enhance angiogenesis in rats. Am. J. Transl. Res. 8, 4289–4299. 27830012 PMC5095321

[B107] Zou X. Kwon S. H. Jiang K. Ferguson C. M. Puranik A. S. Zhu X.. (2018). Renal scattered tubular-like cells confer protective effects in the stenotic murine kidney mediated by release of extracellular vesicles. Sci. Rep. 8:1263. 10.1038/s41598-018-19750-y 29352176 PMC5775303

[B108] Zou X. Zhang G. Cheng Z. Yin D. Du T. Ju G.. (2014). Microvesicles derived from human Wharton's Jelly mesenchymal stromal cells ameliorate renal ischemia-reperfusion injury in rats by suppressing CX3CL1. Stem Cell Res. Ther. 5:40. 10.1186/scrt428 24646750 PMC4055103

